# Formoterol Reduces the Pro‐Inflammatory Phenotype by Enhancing the Activity of Glutaminase in Monocyte‐Derived Macrophages in the CVB3‐Induced Viral Myocarditis

**DOI:** 10.1002/iid3.70073

**Published:** 2024-11-27

**Authors:** Quan‐liang Li, Hua‐bao Xie, Ying‐xin Guo, Juan‐fen Li, Jing Qian, Wei‐Feng Wu

**Affiliations:** ^1^ Department of Cardiology The First Affiliated Hospital of Guangxi Medical University Nanning China; ^2^ Collaborative Innovation Centre of Regenerative Medicine and Medical BioResource Development and Application Co‐constructed by the Province and Ministry, Guangxi Medical University Nanning China

**Keywords:** beta‐2 adrenergic receptor, glutaminase activity, Ly6C monocytes, monocyte‐derived cardiac macrophages, myocarditis

## Abstract

**Background:**

Viral myocarditis (VMC) plays a significant role in heart failure, and there is currently a shortage of available targeted treatments. Macrophage phenotype and function are closely associated with the beta‐2 adrenergic receptor (β2‐AR).

**Method:**

This research employed a BALB/c mouse model of VMC generated using Coxsackievirus B3 (CVB3), and the β2‐AR agonist formoterol was administered as treatment. A bioinformatic analysis was conducted to identify the β2‐AR in CCR2^+^MHCII^high^ monocyte‐derived macrophages (MoMFs). Echocardiography and histopathological assessments were utilized to evaluate cardiac function and inflammation. The enzymatic activity of glutaminase (GLS) was quantified. Flow cytometry was employed to characterize the phenotype and function of the macrophages.

**Result:**

Our study revealed that formoterol treatment effectively mitigated cardiac inflammation and fibrosis, improved cardiac function, and prolonged survival compared to the VMC group. Formoterol reduced the infiltration of CCR2^+^MHCII^high^ MoMFs in the heart, inhibited M1 phenotypic expression and activity, and reduced the percentage of Ly6C^high^ monocytes in circulation. Additionally, formoterol stimulated M2 phenotypic expression and activity and increased the percentage of Ly6C^low^ monocytes in circulation. Additionally, the combination of NICB3344, a C‐C motif chemokine receptor 2 inhibitor, with formoterol did not exhibit synergistic effects on reducing cardiac pathological scores or enhancing cardiac function. In vitro studies involving the use of lipopolysaccharide (LPS)‐induced bone marrow‐derived macrophages, revealed the ability of formoterol to suppress the M1 phenotype and functions induced by LPS while promoting the M2 phenotype and functions. Nevertheless, the observed effects were negated by the introduction of the GLS inhibitor BPTES.

**Conclusion:**

Formoterol potentially serves as a significant metabolic regulator in the differentiation process of cardiac MoMFs, influencing this process by controlling GLS activity. Targeting β2‐AR exhibits potential as an effective approach for managing VMC. It is essential to acknowledge that these findings were derived under specific experimental conditions, with the current conclusions predominantly based on animal models. Future research is necessary to further investigate the feasibility of formoterol in clinical practice.

## Graphical abstract

Notably, the manuscript delineates how formoterol shifts MoMFs from a pro‐inflammatory M1 phenotype to a reparative M2 phenotype, a process mediated by increased glutaminase activity. This mechanism highlights a novel aspect of macrophage function regulation, which could be pivotal for developing new treatments for VMC. Furthermore, our results suggest that targeting β2‐AR could be an effective approach to manage and treat VMC, presenting a significant step forward in the pharmacological management of this disease.

## 1 Introduction

Viral myocarditis (VMC) is defined as heart muscle inflammation caused by viral invasion and is often caused by Coxsackievirus B (CVB), a common trigger of acute myocarditis in children and young adults [1]. It is characterized by myocardial inflammation, which results in acute myocarditis, dilated cardiomyopathy, and congestive heart failure [2]. The pathogenesis of VMC is believed to involve both direct viral effects and the resulting immune response [3]. Monocytes and macrophages play a crucial role in the acute inflammation and fibrosis linked to VMC [4]. Nevertheless, the precise mechanisms through which monocytes and macrophages facilitate disease progression in VMC remain unclear.

Acute myocarditis often leads to substantial damage to the heart muscle and impairment of adrenergic nerve function [5], with the sympathetic nervous system mainly controlling immune system activity through the β2‐adrenergic receptor (β2‐AR) [6]. Multiple studies have illustrated the anti‐inflammatory properties of monocyte/macrophage β2‐AR agonists across various disease states. In a study by Hyunjin Noh and colleagues [7], terbutaline inhibited the activation of peripheral blood mononuclear cells in diabetic rats, reduced the number of C‐C motif chemokine receptor 2 (CCR2) monocytes, and decreased the levels of tumor necrosis factor‐alpha (TNF‐α). Additionally, Lechtenberg et al. [8] reported that clenbuterol attenuated the inflammatory response of microglia and monocyte‐derived macrophages (MoMFs) following cerebral ischemia. Formoterol reduced macrophage infiltration in a rat model of acute pulmonary inflammation, alleviating of lung inflammation [9]. Furthermore, in models of endotoxaemia and acute lung injury, β2‐AR agonists enhance survival rates by modulating the M2 polarization of macrophages [10]. Consequently, β2‐AR agonists exhibit anti‐inflammatory effects on various diseases through the inhibition of monocyte/macrophage activation, inflammatory responses, and shifts in the M1/M2 phenotype.

Different subtypes of cardiac macrophages can be identified based on CCR2 and major histocompatibility complex class II (MHCII) expression levels. CCR2^−^ macrophages, which originate from embryonic sources, are involved in repair and anti‐inflammatory processes, whereas CCR2^+^ macrophages, which are derived from bone marrow monocytes, are recruited during cardiac injury and replenished by the recruitment of Ly6C^high^ monocytes from the bloodstream. CCR2^+^MHCII^high^ MoMFs are linked to pro‐inflammatory reactions [11, 12, 13]. In VMC, macrophages, which are crucial inflammatory cells, are abundant in cardiac tissue 3 days after coxsackievirus B3 (CVB3) infection [14]. Additionally, as the predominant immune cell subset within the heart, macrophages exhibit notable adaptability and versatility in response to diverse pathological stimuli [15, 16]. Nevertheless, there is currently a lack of research investigating the potential of β2‐AR agonists in VMC to reduce the infiltration of cardiac CCR2^+^MHCII^high^ macrophage subpopulations and attenuate their pro‐inflammatory activities to ameliorate inflammation.

Recent research has placed a growing emphasis on the influence of cellular metabolic pathways on modulating the flexibility of immune cell destinies. In a study by Kong et al. [17, 18], nuclear magnetic resonance metabolomic analyses were performed on serum and myocardial tissue samples obtained from mice at various stages of VMC and DCM. The results indicated a disruption in glutamate and glutamine metabolism pathways compared to healthy control mice. Additionally, investigations have demonstrated that increased glutamine metabolism has the potential to drive macrophage differentiation towards the M2 phenotype. Endothelial cells release Rspondin3, stimulating glutamine breakdown and mitochondrial respiration in interstitial macrophages, causing a shift towards an M2 anti‐inflammatory state and ultimately decreasing lung inflammation [19]. Conversely, decreased activity in the glutamine metabolic pathway induces macrophage polarization towards the M1 phenotype. Tumor‐associated macrophages within the tumor microenvironment compete with tumor cells for nutrients. Targeting the glutamine metabolism of TAMs can shift these cells from an M2 to an M1 pro‐inflammatory phenotype, enhancing T‐cell immune responses and slowing tumor growth [20, 21]. In an experiment simulating LPS‐induced sepsis, the use of glutaminase (GLS) blockers reduced survival rates in septic mice and promoted the shift of macrophages towards an M1 inflammatory state by inhibiting GLS activity in a laboratory setting [22]. Hence, the relationship between macrophage glutamine metabolism and functional phenotypes is significant. The inhibition of the glutamine metabolic pathway in macrophages promote the transition towards an M1 pro‐inflammatory phenotype, with GLS playing a pivotal role in this process. Nevertheless, alterations in GLS activity in bone marrow‐derived macrophages (BMDMs) during inflammatory conditions, as well as the potential impact of β2‐AR agonists on GLS activity in modulating the phenotype and function of BMDMs, remain unexplored in the current literature.

The limited efficacy of drug treatments and associated mortality rates in acute VMC make it imperative to identify therapeutic agents. This study investigated the role and mechanism of formoterol in acute VMC. Research on the reduction in β2‐AR levels in MoMFs using single‐cell sequencing data from a mouse model of VMC and identification using flow cytometry analysis in VMC mice has been performed. Subsequently, a study was performed to analyze how the β2‐AR agonist formoterol affects the polarization and function of the CCR2^+^MHCII^high^ MoMF subpopulation in the early stage of VMC in live subjects. Additionally, our in vitro results demonstrated that formoterol inhibits LPS‐induced M1 polarization of macrophages by augmenting GLS activity. These findings offer a new avenue for the treatment of VMC.

## 2 Materials and Methods

### 2.1 Animals

Six‐week‐old male BALB/c mice were procured from Beijing SiPeiFu Biotechnology Co. Ltd. The mice were kept in a sterile environment at the Medical Experimental Animal Center of Guangxi Medical University. A mouse model for VMC was created by injecting 0.1 mL of 10^3^ PFU of passaged CVB3 intraperitoneally, as described in previous research [14]. An equal amount of PBS was administered to the control group. Mice were euthanized 1 week and 2 weeks after being infected with CVB3. All experimental procedures conformed with the guidelines of the Ethics Committee of First Affiliated Hospital of Guangxi Medical University, and permission from Ethics Committee belonging to First Affiliated Hospital of Guangxi Medical University was granted (202306013).

### 2.2 Cell Culture

Bone marrow cells were extracted and cultured in vitro following established methods reported in the literature [23]. Briefly, in a sterile environment, the femurs and tibias of mice were collected and consecutively treated with PBS containing penicillin and streptomycin, followed by 75% ethanol for 5 min. Subsequently, the bones were rinsed three times with sterile PBS. The marrow cells were extracted from the cavity using a sterile syringe filled with chilled DMEM and then transferred to a sterile tube for centrifugation. After dispersing the cells, they were passed through a 70‐μm strainer and then centrifuged. After the pellet was resuspended, it was mixed thoroughly in full DMEM containing 20‐ng/mL M‐CSF (HY‐P7085, MCE, USA) and 10% FBS and then transferred to 6‐well plates for seeding. The cultures were then incubated in a 5% CO_2_, 37°C environment with medium changes every 3–4 days and utilized for further experiments on Day 7.

### 2.3 Bioinformatic Analysis

Data from single‐cell sequencing GSE174458 [24] and RNA transcriptome high‐throughput sequencing GSE99296 [22] were acquired from the Gene Expression Omnibus (GEO) database (https://ncbi.nlm.nih.gov/geo/). The GSE174458 data set consists of samples from two control groups and two VMC samples, whereas GSE99296 includes samples from two control groups and two LPS‐stimulated bone marrow‐derived macrophage groups. To analyze the GSE174458 data set, we initially created an analytical object using the Seurat R package [25] and subsequently performed cluster analysis on the cells. Next, we conducted cell type annotations within individual clusters by leveraging gene expression correlations with the Immune Genome Project (ImmGen) database [26] through the utilization of the SingleR R package [27]. Next, we analyzed CCR2^+^MHCII^high^ MoMFs by labeling cells with specific markers and examining gene expression patterns in the identified cell groups. In the fourth step of the study, we analyzed the β2‐AR expression on CCR2^+^MHCII^high^ MoMFs. Subsequently, for the GSE99296 transcriptome data set, differential gene analysis was initially performed using the DESeq2 package. The DEGs were subsequently subjected to Gene Ontology (GO) enrichment analysis, Kyoto Encyclopedia of Genes and Genomes (KEGG) enrichment analysis, and gene set enrichment analysis (GSEA) and were subsequently visualized using a freely accessible bioinformatics analysis tool [28].

### 2.4 Drug Intervention

From Day 1 to Day 14 after infection with CVB3, mice with VMC were administered intraperitoneal injections of formoterol (HY‐B0010, MCE, USA) at a dosage of 0.3 mg/kg. The regimen was consistent with previously published studies [29] and has been shown to be safe for mice. Similarly, following approved procedures [30], the CCR2 blocker INCB3344 (HY‐50674, MCE, USA) was administered at a dose of 30 mg/kg/day through intraperitoneal injection to VMC mice starting on Day 1 and continuing until Day 14 after CVB3 infection. A 1% DMSO solution served as the solvent control. In vitro‐cultured BMDMs were identified as mostly macrophages (> 90%) by Day 7 using flow cytometry analysis, then treated with 100‐ng/mL LPS. Before LPS stimulation, cells were pretreated with either 10^−^^6^ M formoterol [31] or 10‐mM GLS inhibitor BPTES (HY‐12683, MCE, USA) [22] for in vitro intervention for 1 h, with 0.1% DMSO serving as the solvent control, as described in previous studies. The cells were harvested for additional analysis 12‐h postintervention.

### 2.5 Echocardiograph

All mice were subjected to echocardiographic assessment on Days 7 and 14 following infection with CVB3 using transthoracic echocardiography. Mice were anaesthetized using a 1.25% Avertin solution at a dose of 0.1‐mL per 10 g of body weight. After hair removal, the chest was imaged using a 22‐Hz ultrasound probe from the MylabSat system to obtain standard long‐axis and short‐axis views. Methods described in previous studies [32] were used to measure the left ventricular ejection fraction (LVEF) and left ventricular fractional shortening (LVFS).

### 2.6 Histopathology

After removal, the hearts of the mice were placed in 4% paraformaldehyde for 24 h and then embedded in paraffin. Afterwards, 5‐μm‐thick sections were obtained from the heart tissues that were embedded and then stained with haematoxylin and eosin (H&E) for examining pathological changes under a microscope. The severity of VMC was then evaluated by determining myocardial pathology scores using methodology documented in prior literature [33]. The severity of myocarditis was assessed using a grading scale ranging from 0 to 4, which corresponded to varying degrees of cardiac inflammation as follows: < 25% cardiac inflammation, 25%–50% cardiac inflammation, 50%–75% cardiac inflammation, and > 75% cardiac inflammation. Masson's trichrome stain was used to measure the level of fibrosis in the heart muscle. The VMC severity and extent of fibrosis were evaluated using an Olympus microscope at 200× magnification, and a random selection of five fields from each slide was used to calculate myocardial interstitial fibrosis using ImageJ V.1.52a (Wayne Rasband, New York, USA) according to previous study [34].

### 2.7 GLS Activity Assay

Previous studies [35] evaluated GLS function in different groups of BMDMs using a GLS activity test kit (BC1455, Solarbio, China) following the instructions provided by the manufacturer. Briefly, cell lysates were mixed with the samples to be isolated and then ultrasonicated in an ice bath to disrupt the cells, followed by centrifugation at 4°C and 12,000 × g for 15 min. The resulting supernatant was then mixed with the working solution. After incubating for 30 min at ambient temperature, the absorbance was recorded at 630 nm using a spectrophotometer. The resulting value signifies that one unit of enzyme activity is defined by the enzyme's capacity to catalyze the production of 1 μmol of NH3‐N per mg of protein per hour at 37°C.

### 2.8 Flow Cytometry

Heart tissue single‐cell suspensions for flow cytometry analysis were generated according to established protocols outlined in the literature [36]. Before isolation, the hearts were perfused with PBS and subsequently mechanically dissociated into 2‐mm thick fragments in precooled PBS. Following centrifugation at 50 g for 2 min, the supernatant was removed. The tissue pieces were subsequently broken down with a mixture of collagenase II, hyaluronidase, and DNase in HBSS buffer and incubated on a shaker at 37°C for 40 min. Following the digestion process, the cardiac tissue suspension was combined at a 1:1 ratio with a precooled PBS termination solution containing 2% FBS and 2‐μM EDTA to stop the digestion. Subsequently, the digestion was stopped, and the resulting mixture was filtered through a 40‐μm strainer and centrifuged at 350 g for 5 min, after which the supernatant was discarded. After centrifugation, the cells were lysed using a solution to remove red blood cells for 3 min, after which PBS was added to stop the lysis. After further centrifugation at 350 g for 5 min, the cells were reconstituted in 100 μl of PBS for subsequent use. BMDMs were produced as single‐cell suspensions for each group in the experiment. Mouse peripheral blood mononuclear cells were obtained from mice using a kit for separating mouse peripheral blood mononuclear cells (P6340, Solarbio, China) according to the instructions provided by the manufacturer. Before staining, all the samples were incubated with anti‐CD16/CD32 at 4°C in the dark for 15 min to prevent nonspecific binding. Heart cells, BMDM cells, and blood mononuclear cells were labeled with Fixable Viability Dye BV510 (eBioscience, USA), anti‐CD45 APC/Cyanine7 (30‐F11, Biolegend, USA), anti‐CD64 PerCP/Cyanine5.5 (X54‐5/7.1, Biolegend, USA), anti‐F4/80 BV421 (T45‐2342, Biolegend, USA), anti‐MHC Class II FITC (M5/114.15.2, eBioscience, USA), anti‐CCR2 PE/Cyanine7 (SA203G11, Biolegend, USA), anti‐β2‐AR PE/Cyanine7 (orb917503, BIORBYT, UK), anti‐Ly6C APC (HK1.4, Invitrogen, USA), anti‐Ly6G FITC (1A8, BD, USA), anti‐iNOS PE (CXNFT, eBioscience, USA), anti‐CD206 PE‐Cyanine7 (MR6F3, eBioscience, USA), anti‐TNF‐α AF700 (MP6‐XT22, Biolegend, USA), anti‐CCL2 APC (2H5, Biolegend, USA), anti‐CD86 PE (GL1, Invitrogen, USA), anti‐IL‐10 BV421 (JES5‐16E3, BD, USA), anti‐CD11b FITC (M1/70, Biolegend, USA), and anti‐F4/80 PerCP/Cyanine5.5 (BM8, Invitrogen, USA). CD45^+^CD64^+^F4/80^+^ cells were identified as cardiac macrophages. Cardiac MoMFs were defined as CD45^+^CD64^+^F4/80^+^MHCII^high^CCR2^+^ cells. In this group, the M1 phenotype was identified as CD45^+^CD64^+^F4/80^+^MHCII^high^CCR2^+^iNOS^+^ cells, whereas the M2 phenotype was described as CD45^+^CD64^+^F4/80^+^MHCII^high^CCR2^+^CD206^+^ cells. To stain the BMDM single‐cell suspensions, the following antibodies were used: Fixable Viability Dye BV510 (eBioscience, USA), anti‐CD11b BB70 (M1/70, BD, USA), anti‐F4/80 BV421 (T45‐2342, Biolegend, USA), anti‐CD86 APC (PO3, Biolegend, USA), and anti‐CD206 PE‐Cyanine7 (MR6F3, eBioscience, USA). CD11b^+^F4/80^+^ cells were identified as bone marrow macrophages. CD11b^+^F4/80^+^CD86^+^ cells were classified as M1, whereas CD11b^+^F4/80^+^CD206^+^ cells were identified as M2. Peripheral blood mononuclear cells were defined as CD45^+^CD11b^+^Ly6G^‐^Ly6C^+^ cells, which were further subdivided based on Ly6C expression levels into Ly6C^high^ and Ly6C^low^ populations. A FACSCanto Flow Cytometer was used to analyze all the samples. Analysis of flow cytometry data was conducted with FlowJo V10 software (BD Biosciences).

### 2.9 Statistical Analysis

The data are presented as the mean ± SEs and were analyzed using SPSS software (version 24.0). Student's *t*‐test was used to compare two groups, and one‐way analysis of variance (ANOVA) with Tukey's post hoc test (using the Holm correction) was used for comparisons of more than two groups. Significance levels are indicated by *p < 0.05, **p < 0.01, ***p < 0.001, and ****p < 0.0001.

## 3 Results

### 3.1 Reduced β2‐AR Expression on MoMFs in VMC

**Figure 1:**
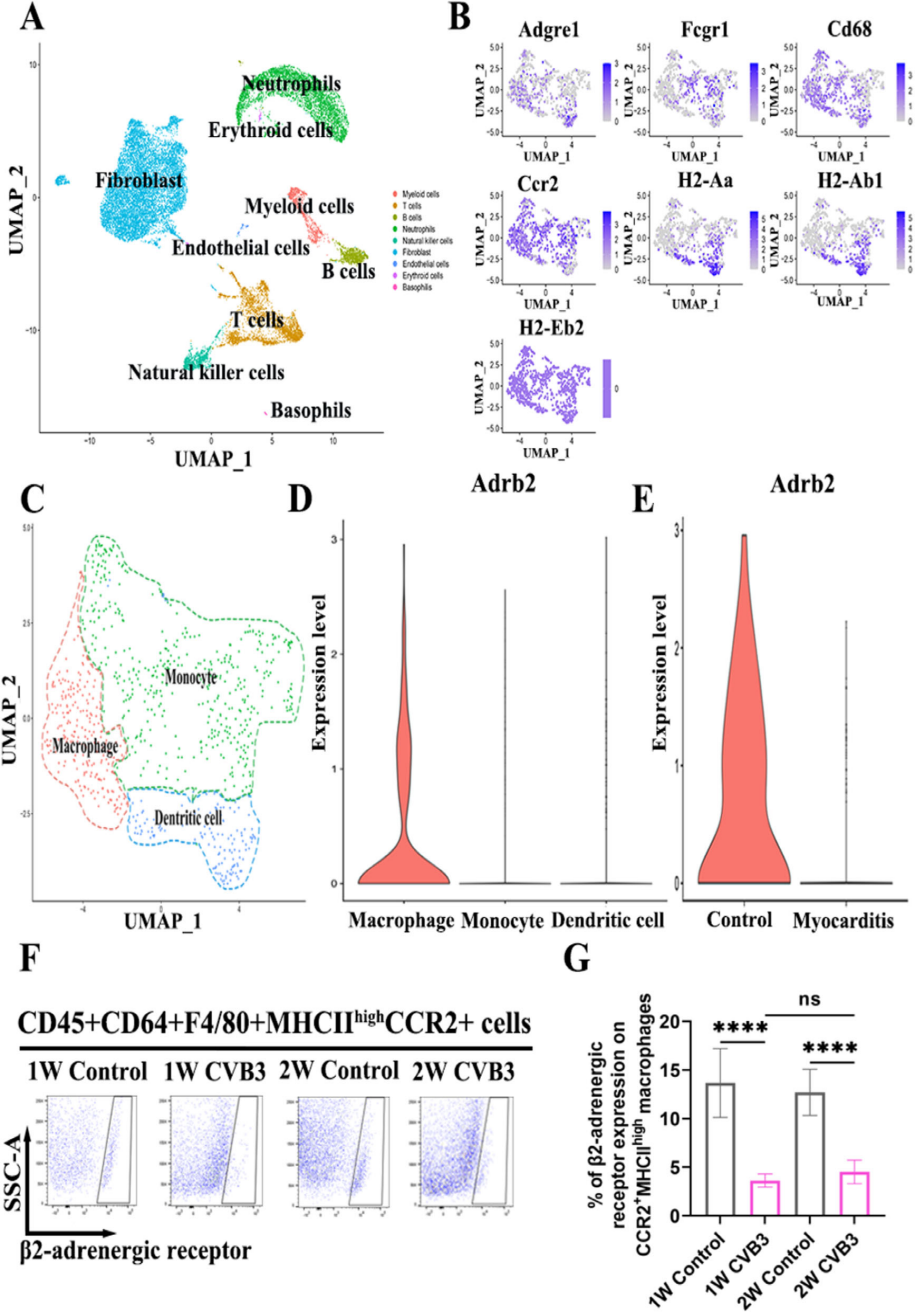
β2‐AR expression on CCR2^+^MHCII^high^ MoMFs. (A) UMAP of cluster annotations. (B) UMAP plots of different markers of cell types in annotated myeloid cells. (C) UMAP of myeloid cell annotations. (D) Violin plot of Adrb2 expression on CCR2^+^MHCII^high^ macrophages, monocytes, and dendritic cells. (E) Violin plot of Adrb2 expression on CCR2^+^MHCII^high^ macrophages in the control and myocarditis groups. (F) Flow cytometry analysis of β2‐adrenergic receptor expression on CD45^+^CD64^+^F4/80^+^CCR2^+^MHCII^high^ MoMFs from myocarditis mice 1 week and 2 weeks after CVB3 infection and from control mice. (G) Statistical histogram of the flow cytometry results (*n* = 6/group). The data are presented as means ± SEMs. *p< 0.05, **p< 0.01, ***p< 0.001, ****p< 0.0001. β2‐AR, adrenergic receptor; CCR2, C‐C chemokine receptor type 2; MHCII, major histocompatibility complex class II; MoMFs, monocyte‐derived macrophages; UMAP, uniform manifold approximation and projection; SEM, standard error and mean.

During the onset of VMC, MoMFs, which are integral to the innate immune response, play a pivotal role. To investigate the expression dynamics of β2‐AR in CCR2^+^MHCII^high^ MoMFs during the pathogenesis of VMC, we reanalyzed the single‐cell data set GSE99296 from mice in the acute phase of the disease. Uniform manifold approximation and projection (UMAP) clustering and annotation revealed the predominance of nine cell types, including myeloid cells, T cells, B cells, neutrophils, natural killer cells, fibroblasts, endothelial cells, erythroid cells, and basophils, in cardiac single‐cell suspensions from mice with VMC (Figure 1A). To further study myeloid cell subtypes, we performed clustering and cell annotation specifically for myeloid cells, identifying three subtypes: macrophages, monocytes, and antigen‐presenting cells (Figure 1C). The expression of the MoMF markers F4/80, CCR2, and MHCII and other surface markers across these subtypes was then illustrated using a UMAP plot (Figure 1B). Subsequently, we compared the expression of the Adrb2 gene, which encodes β2‐AR, across macrophages, monocytes, and antigen‐presenting cells and observed increased expression on macrophages compared with other cell types (Figure 1D). Further analysis of Adrb2 expression on CCR2^+^MHCII^high^ MoMFs in the myocarditis‐afflicted and normal control groups revealed a decrease in Adrb2 expression in the myocarditis group (Figure 1E). Finally, flow cytometric analysis of β2‐AR expression on MoMFs from the hearts of mice with VMC showed a reduction in β2‐AR expression both during the initial inflammatory phase at week one and at the inflammation peak in week two compared to those in the normal control group and there was no difference in β2‐AR expression on MoMFs between CVB3 group at week one and at the inflammation peak in week two (Figure 1F,G). Overall, these results indicate that reduced β2‐AR expression on cardiac MoMFs could play a role in the development of VMC.

### 3.2 Formoterol Mitigated the Severity of VMC

**Figure 2:**
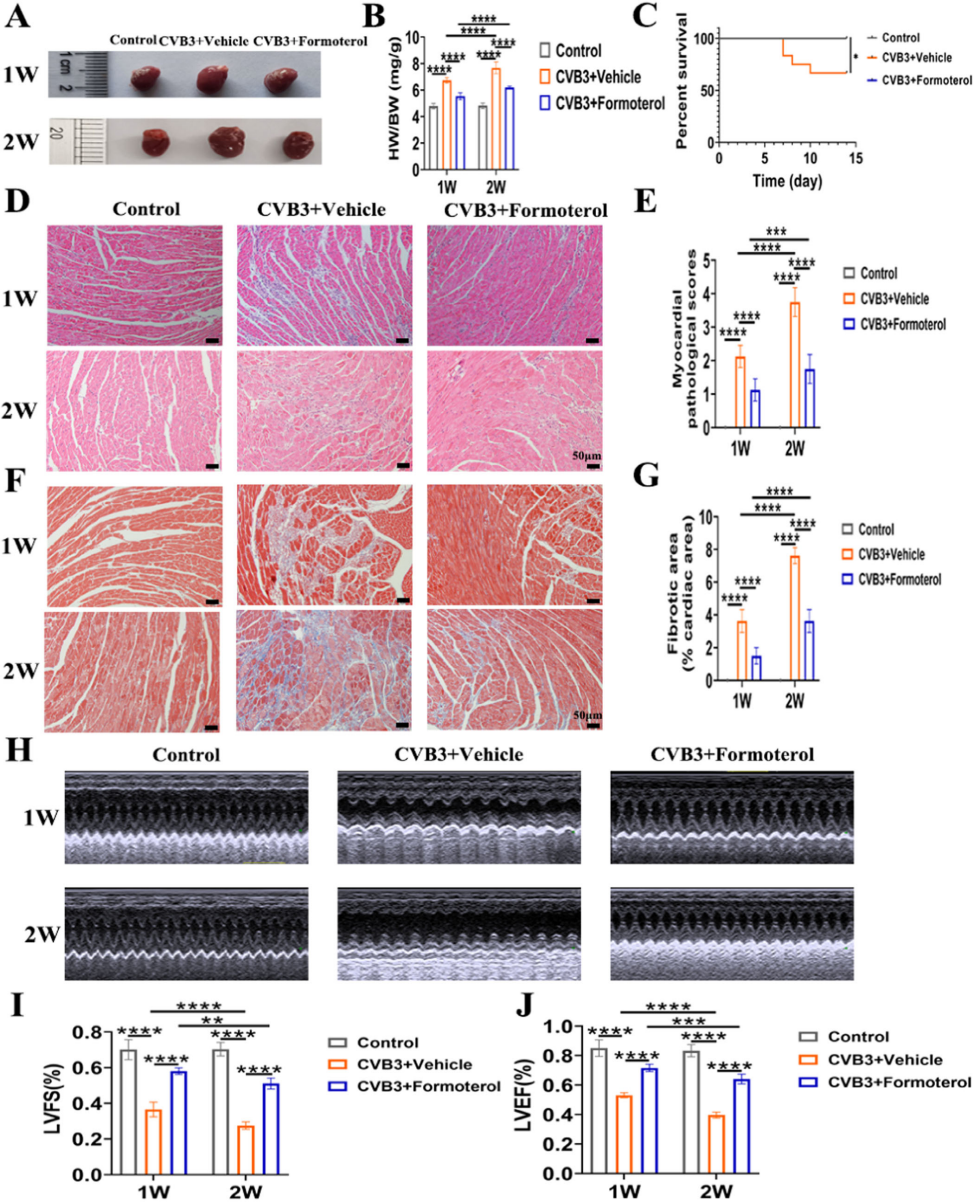
Formoterol attenuates the severity of viral myocarditis. (A) Representative images of mouse hearts in each group after CVB3 infection for 1 week or 2 weeks. (B) The HW/BW ratio of each group after CVB3 infection or 2 weeks (*n* = 8–10/group). (C) Kaplan–Meier survival curves of mice in different treatment groups (*n* = 12/group). (D) Representative H&E staining images of each group after CVB3 infection for 1 week and 2 weeks (original magnification, 200×; black scale bars = 50 µm). (E) Statistical results of myocardial pathological score of each group after CVB3 infection 1 week and 2 weeks (*n* = 8/group). (F) Representative Masson's trichrome staining images of each group after CVB3 infection 1 week and 2 weeks (original magnification, 200×; black scale bars = 50 µm). (G) Statistical analysis of the cardiac collogen volume fractions of each group after CVB3 infection for 1 week and 2 weeks (*n* = 8/group). (H) Representative cardiac ultrasound images of mice in each group after CVB3 infection for 1 week or 2 weeks. (I and J) Statistical analysis of the LVFS (I) and LVEF (J) of each group after CVB3 infection for 1 week or 2 weeks (*n* = 8/group). The data are presented as the means ± SEMs. *p < 0.05, **p < 0.01, ***p < 0.001, ****p < 0.0001. CVB3, coxsackievirus B3; LVFS, left ventricular fractional shortening; LVEF, left ventricular ejection fraction; H&E, haematoxylin and eosin; HW/BW, heart weight/body weight; SEM, standard error and mean.

To determine the role of β2‐AR in the development of VMC, mice were administered formoterol, a β2‐AR agonist, during the initial inflammation phase in the first week and at the height of inflammation in the second week of VMC. Heart weight to body weight (HW/BW) ratio, myocardial pathological scores, and fibrotic area were significantly elevated in CVB3 model group at both weeks 1 and 2, whereas left ventricular fractional shortening (LVFS) and left ventricular ejection fraction (LVEF) were significantly reduced in comparison to the normal control group; when compared to the CVB3 model group at week 1, the HW/BW ratio, myocardial pathological scores, and fibrotic area in the model group at week 2 exhibited further increases, while LVFS and LVEF continued to decline (Figure 2B,E,G,I,H). These findings suggest that the establishment and progression of the CVB3 model are consistent with the traditional changes observed in acute VMC. Compared with control mice treated with solvent, mice treated with formoterol showed decreased cardiac swelling and a lower HW/BW ratio in both the first and second weeks (Figure 2A,B). Survival also increased in formoterol‐treated mice (Figure 2C). Examinations of tissue samples with haematoxylin and eosin (H&E) staining and Masson's trichrome staining revealed a reduction in myocardial inflammation (Figure 2D,E) and fibrosis (Figure 2F,G) in the hearts of mice treated with formoterol. Consistent with these results, echocardiographic evaluations revealed enhancements in LVFS and LVEF among mice receiving treatment (Figure 2H–J). These findings indicate that β2‐AR may have a protective effect, making its stimulation a possible target for treating VMC.

### 3.3 Formoterol Reduced CCR2^+^MHCII^high^ MoMF Infiltration

**Figure 3:**
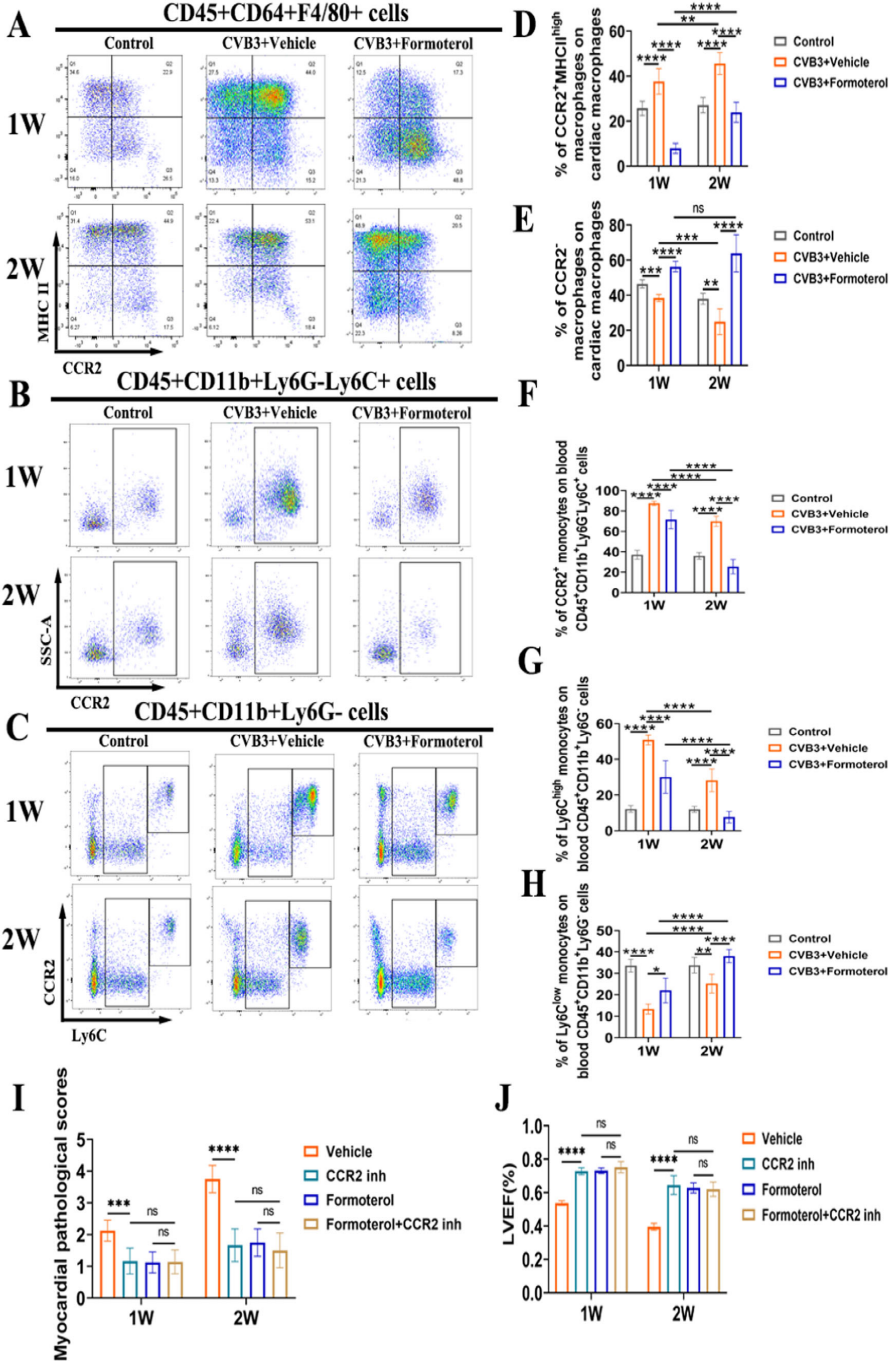
The effect of formoterol on the frequencies of cardiac macrophages and blood monocytes. (A) Representative flow cytometry images of cardiac macrophage subsets among cardiac CD45^+^CD64^+^F4/80^+^ cells in the control group, CVB3^+^vehicle group and CVB3^+^formoterol group after CVB3 infection for 1 week or 2 weeks. (B) Representative flow cytometry images of CCR2^+^ monocytes among blood CD45^+^CD11b^+^Ly6G^‐^Ly6C^+^ cells in the control group, CVB3^+^vehicle group and CVB3^+^formoterol group after CVB3 infection for 1 week or 2 weeks. (C) Representative flow cytometry images of Ly6C^high^ monocytes and Ly6C^low^ monocytes among blood CD45^+^CD11b^+^Ly6G^−^ cells in the control group, CVB3^+^vehicle group and CVB3^+^formoterol group after CVB3 infection for 1 week or 2 weeks. (D, E) Statistical results of flow cytometric analysis of the frequencies of CCR2^+^MHCII^high^ MoMFs (D) and CCR2‐ macrophages (E) among cardiac macrophages in the control group, CVB3^+^vehicle group and CVB3^+^formoterol group after CVB3 infection for 1week or 2 weeks (*n* = 6/group). (F) Statistical results of flow cytometric analysis on the frequencies of CCR2^+^ monocytes among blood CD45^+^CD11b^+^Ly6G‐Ly6C^+^ cells in the control group, CVB3^+^vehicle group and CVB3^+^formoterol group after CVB3 infection for 1 week or 2 weeks (*n* = 6/group). (G, H) Statistical results of flow cytometric analysis of the frequencies of Ly6C^high^ monocytes (G) and Ly6C^low^ monocytes (H) among blood CD45^+^CD11b^+^Ly6G‐ cells in the control group, CVB3^+^vehicle group and CVB3^+^formoterol group after CVB3 infection for 1 week or 2 weeks (*n* = 6/group). (I, J) Myocardial pathological scores (I) and LVEF (J) of CVB3^−^ and CVB3^+^Formoterol‐treated mice administered DMSO or a CCR2 inhibitor after 1 week and 2 weeks (*n* = 6/group). The data are presented as the means ± SEMs. *p < 0.05, **p < 0.01, ***p < 0.001, ****p < 0.0001. CCR2, C‐C chemokine receptor type 2; CVB3, coxsackievirus B3; MHCII, major histocompatibility complex class II; MoMFs, monocyte‐derived macrophages; LVEF, left ventricular ejection fraction; SEM, standard error of the mean.

To assess the impact of formoterol on MoMF infiltration in the heart during the early and peak stages of inflammation in the VMC, cardiac tissues and peripheral blood were collected for flow cytometric analysis both during the first week of early inflammation and the second week of peak inflammation. In comparison to the normal control group, the frequencies of MoMFs in the heart and inflammatory monocytes (CCR2^+^ monocytes and Ly6C^high^ monocytes) in the blood were elevated in CVB3 model group at weeks 1 and 2, while CCR2^‐^ macrophages in the heart and reparative Ly6C^low^ monocytes in the blood were decreased; when compared to the CVB3 model group at week 1, the MoMFs in the heart and Ly6C^low^ monocytes in the blood were increased in the model group at week 2, whereas the CCR2^‐^ macrophages in the heart and the inflammatory monocytes (CCR2^+^ monocytes and Ly6C^high^ monocytes) in the blood were decreased (Figure 3A–H). The result indicate that inflammation exacerbates during the second week, potentially attributable to the continued depletion of CCR2^−^macrophages in the heart, the infiltration of blood pro‐inflammatory monocytes into the cardiac tissue, and the subsequent increase in MoMFs within the cardiac tissue. Compared to solvent‐treated VMC mice, mice treated with formoterol exhibited reduced infiltration of CCR2^+^MHCII^high^ MoMFs in the heart during both the first and second weeks, and an increase in CCR2^‐^ resident cardiac macrophages (Figure 3A,D,E). Furthermore, a decrease in peripheral blood CCR2^+^ monocytes was observed in formoterol‐treated mice (Figure 3B,F), along with a decrease in inflammation‐related Ly6C^high^ monocytes and an increase in the repair‐associated Ly6C^low^ monocytes (Figure 3C,G,H). Finally, to investigate whether formoterol acts through bone marrow‐derived CCR2^+^ monocytes/macrophages, we treated cell with a CCR2 antagonist. The results revealed that the use of the CCR2 antagonist alone, without formoterol, reduced cardiac inflammation and improved cardiac function compared to those in solvent‐treated group. A combined effect was not observed when formoterol was combined with the CCR2 antagonist, and the groups treated with only formoterol did not significantly differ from those treated with both agents (Figure 3I,J). These findings suggest that formoterol reduces CCR2^+^MHCII^high^ MoMF infiltration during the early and peak phases of VMC, primarily by decreasing the infiltration of bone marrow‐derived CCR2^+^ monocytes/macrophages.

### 3.4 Formoterol Suppressed the Differentiation of CCR2^+^MHCII^High^ MoMFs to the M1 Phenotype While Enhancing Their Transition to the M2 Phenotype

**Figure 4:**
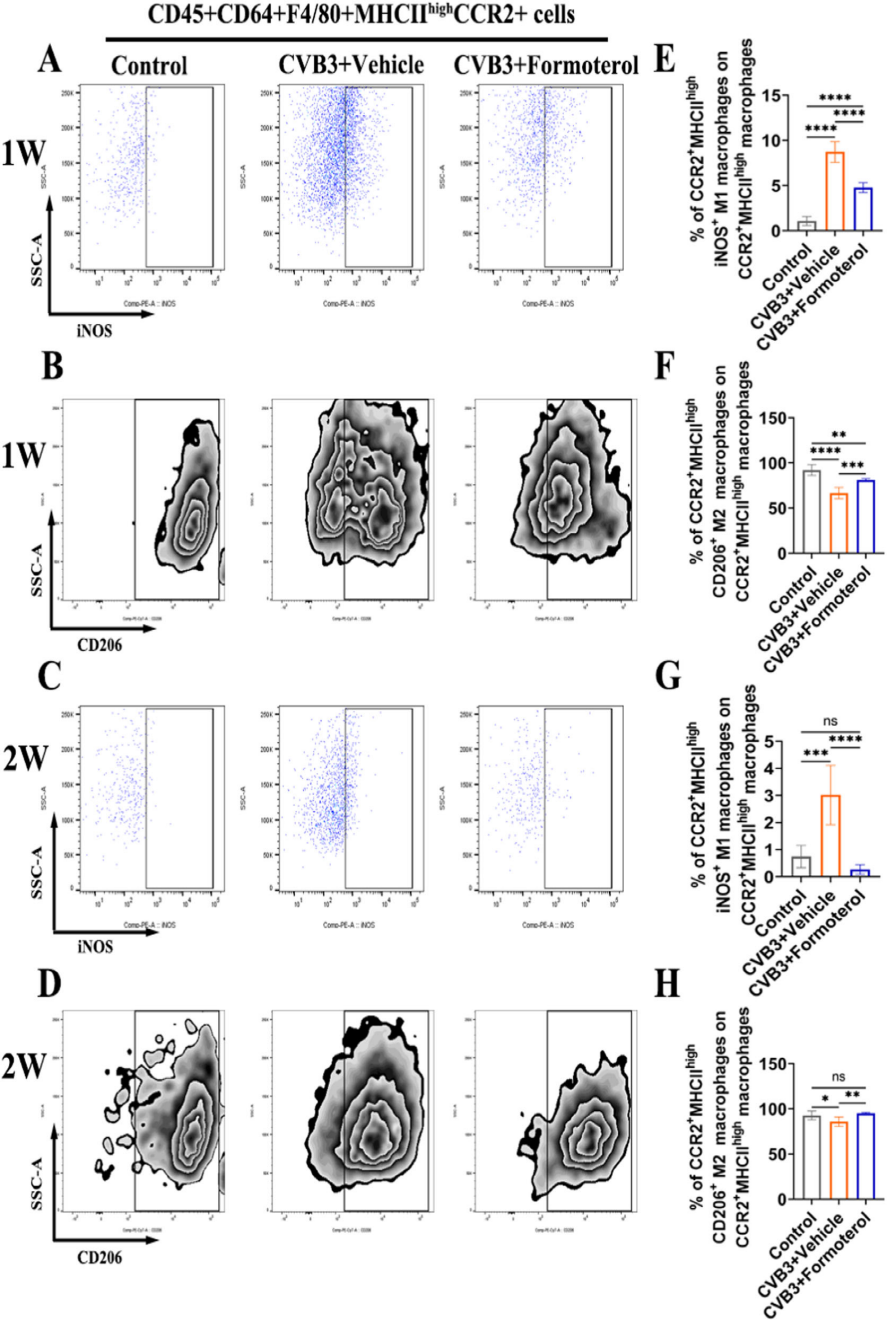
Formoterol promotes cardiac CCR2^+^MHCII^high^ MoMF polarization to the M2 phenotype in BALB/c mice with viral myocarditis. (A–D) Representative flow cytometry images of monocyte‐derived CCR2^+^MHCII^high^iNOS^+^M1 macrophages and CCR2^+^MHCII^high^CD206^+^M2 macrophages in the CD45^+^CD64^+^F4/80^+^CCR2^+^MHCII^high^ macrophages of the viral myocarditis group and control group after CVB3 infection for 1 week (A, B) or 2 weeks (C, D). (E–H) Statistical results of flow cytometric analysis of the frequencies of monocyte‐derived CCR2+MHCII^high^iNOS^+^M1 macrophages and CCR2^+^MHCII^high^CD206^+^M2 macrophages among CD45^+^CD64^+^F4/80^+^CCR2^+^MHCII^high^ macrophages in the control group, CVB3^+^vehicle group and CVB3^+^formoterol group after CVB3 infection for 1 week (E, F) (*n* = 6/group) or 2 weeks (G, H) (*n* = 6/group). The data are presented as the means ± SEMs. *p < 0.05, **p < 0.01, ***p < 0.001, ****p < 0.0001. CCR2, C‐C chemokine receptor type 2; CVB3, coxsackievirus B3; iNOS, inducible nitric oxide synthase; MHCII, major histocompatibility complex class II; MoMFs, monocyte‐derived macrophages; SEM, standard error of the mean.

To explore the effects of formoterol on the polarization of MoMFs, flow cytometric analysis was conducted on cardiac cells both during the early stage of inflammation in the first week and at the peak of inflammation in the second week of VMC. Compared to the solvent, formoterol significantly inhibited the M1 polarization of CCR2^+^MHCII^high^ MoMFs (Figure 4A,E) and promoted M2 polarization during the early stage of VMC inflammation (Figure 4B,F). Similarly, during the peak of inflammation, formoterol continued to suppress M1 polarization (Figure 4C,G) and enhance M2 polarization (Figure 4D,H). These findings indicate that formoterol modulates the polarization of MoMFs towards the M2 phenotype both during the early and peak phases of inflammation in the VMC, thereby alleviating cardiac inflammatory responses.

### 3.5 Formoterol Reduced the Ability of MoMFs to Express Costimulation Signals and Their Ability to Attract Cells, Decreased the Production of Inflammatory Cytokines, and Increased the Release of Anti‐Inflammatory Cytokines

**Figure 5:**
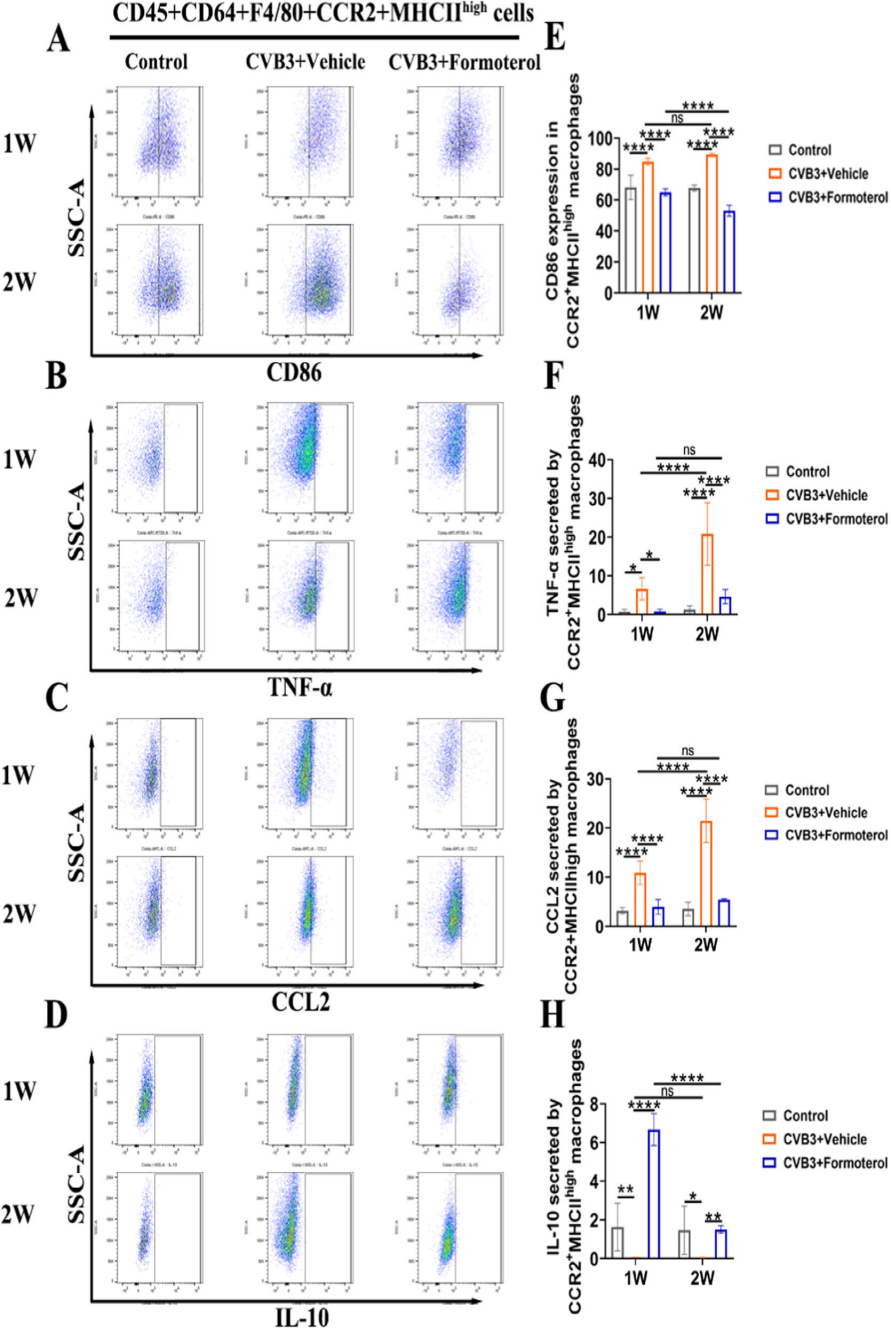
The effect of formoterol on the function of CCR2^+^MHCII^high^ MoMFs in BALB/c mice with viral myocarditis. (A–D) Representative flow cytometry images of CD86 (A), TNF‐α (B), CCL2 (C), and IL‐10 (D) expressed or secreted by CD45^+^CD64^+^F4/80^+^CCR2^+^MHCII^high^ macrophages in the control group, CVB3^+^vehicle group and formoterol group after CVB3 infection for 1 week and 2 weeks. (E–H) Statistical results of flow cytometric analysis of the frequencies of CD86 (E), TNF‐α (F), CCL2 (G), and IL‐10 (H) expressed or secreted by CD45^+^CD64^+^F4/80^+^CCR2^+^MHCII^high^ macrophages in the control group, CVB3^+^vehicle group and CVB3^+^formoterol group after CVB3 infection for 1 week and 2 weeks (*n* = 6/group). The data are presented as the means ± SEMs. *p < 0.05, **p < 0.01, ***p < 0.001, ****p < 0.0001. CCL2, C‐C motif chemokine ligand 2; CCR2, C‐C chemokine receptor type 2; CVB3, coxsackievirus B3; IL‐10, interleukin 10; MHCII, major histocompatibility complex class II; MoMFs, monocyte‐derived macrophages; SEM, standard error of the mean; TNF‐α, tumor necrosis factor‐α.

Flow cytometric analyses were conducted on cardiac cells during the initial week of VMC and at the peak of inflammation in the second week to assess the effects of formoterol on the costimulatory signaling ability, chemotactic function, and secretion of pro‐inflammatory and anti‐inflammatory cytokines in MoMFs. In comparison to the normal control group, the CVB3 model group exhibited elevated CD86 expression in MoMFs and increased secretion of TNF‐α during the first and second weeks, while the secretion of IL‐10 was reduced; When comparing the CVB3 model group at week 1 to week 2, there was a further increase in TNF‐α secretion by MoMFs, with no significant differences observed in CD86 expression and IL‐10 secretion (Figure 5A–H). These findings suggest that the heightened inflammation observed in the second week is linked to the enhanced pro‐inflammatory function of MoMFs. The results showed that formoterol effectively reduced the ability of MoMFs to induce costimulation signals by decreasing CD86 levels (Figure 5A,E), decreasing TNF‐α secretion (Figure 5B,F), decreasing CCL2 expression (Figure 5C,G), and increasing IL‐10 secretion (Figure 5D,H) during early and peak inflammation in the VMC. Notably, there was no significant difference in TNF‐α and CCL2 levels in the formoterol group at week 2 compared to week 1 (Figure 5B,F,C,G). However, the formoterol group at week 2 exhibited a reduction in CD86 expression and an increase in IL‐10 levels relative to the formoterol group at week 1 (Figure 5A,E,D,H). These findings indicate that formoterol helps reduce inflammation in mice with VMC by decreasing the ability of MoMFs to induce costimulation signals and attract cells, decreasing the production of inflammatory cytokines, and increasing the release of anti‐inflammatory cytokines, regardless of the inflammation stage.

### 3.6 Formoterol Enhanced GLS Activity in LPS‐Induced BMDMs In Vitro

**Figure 6:**
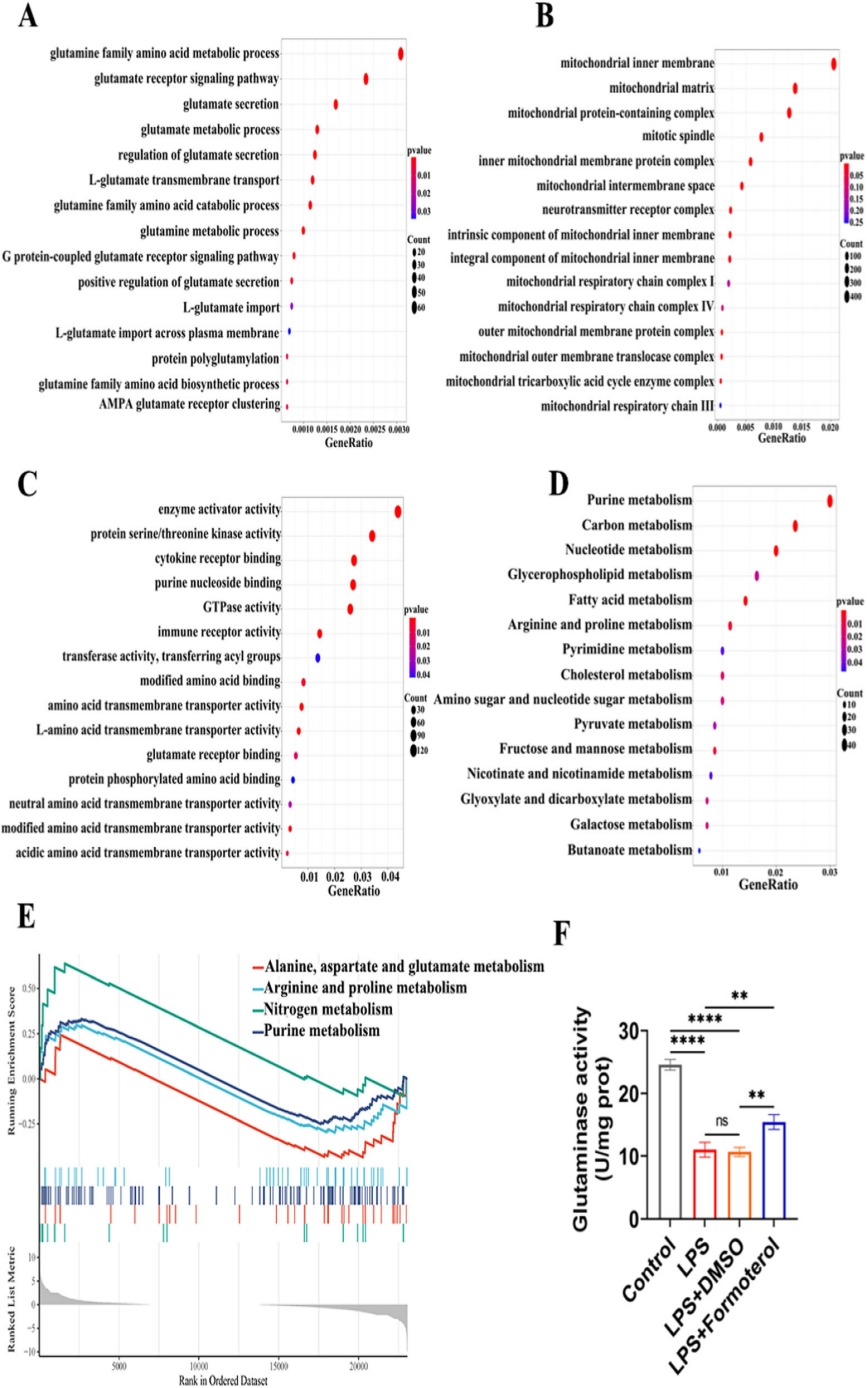
The effect of formoterol on glutaminase activity in LPS‐induced BMDMs. (A–C) The results of GO enrichment analysis are summarized for the following categories: biological process (A), cellular component (B), and molecular function (C). The *y*‐axis indicates functional groups. The *x*‐axis indicates gene ratio. (D) The enrichment analysis of KEGG metabolism pathways. The *y*‐axis indicates metabolic pathways. The *x*‐axis indicates gene ratio. (E) The enrichment analysis of GSEA pathways shows the associated glutamine metabolism pathways. (F) Statistical analysis of the effect of formoterol on glutaminase activity in each group (*n* = 3/group). The data are presented as the means ± SEMs. *p < 0.05, **p < 0.01, ***p < 0.001, ****p < 0.0001. GO, Gene Ontology; GSEA, gene set enrichment analysis; KEGG, Kyoto Encyclopedia of Genes and Genomes; LPS, lipopolysaccharides; SEM, standard error of the mean.

To investigate glutamine‐related metabolic activity in BMDMs under inflammatory conditions, we analyzed the existing GEO data set GSE99296 and conducted in vitro experiments to assess the effects of formoterol on GLS activity in LPS‐induced BMDMs. GO and KEGG enrichment analyses of differentially expressed genes between LPS‐induced BMDMs and normal control cells revealed associations with biological processes related to glutamine metabolism (Figure 6A), mitochondrial respiratory components (Figure 6B), amino acid binding (Figure 6C), and transport functions (Figure 6D). GSEA indicated a decrease in the metabolism of alanine, aspartate, and glutamate (Figure 6E). Our in vitro experiments corroborated these findings; compared to that in the control group, GLS activity was decreased in the LPS‐induced BMDMs, but increased following the administration of formoterol (Figure 6F). These results suggest that GLS activity in BMDMs is reduced under inflammatory conditions and that formoterol can increase GLS activity in LPS‐induced BMDMs.

### 3.7 Formoterol Reduced M1 Polarization and the Release of Inflammatory Cytokines in BMDMs Induced by LPS by Relying on GLS

**Figure 7:**
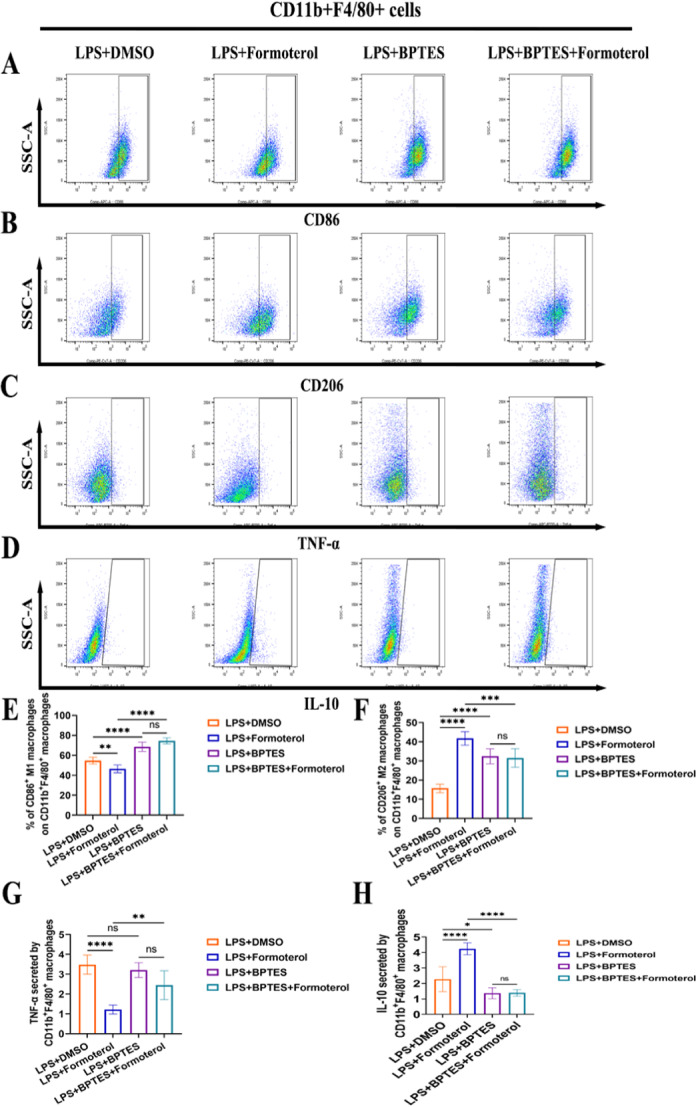
The effect of formoterol on M1 macrophages polarization in LPS‐induced BMDMs is dependent on glutaminase activity. (A–D) Representative flow cytometry images of CD11b^+^F4/80^+^CD86^+^M1 macrophages (A), CD11b^+^F4/80^+^CD206^+^M2 macrophages (B), and TNF‐α (C) and IL‐10 (D) levels in LPS‐induced BMDMs treated with DMSO, Formoterol, BPTES (an inhibitor of glutaminase activity), BPTES^+^formoterol. (E, F) Statistical results of flow cytometric analysis of the frequencies of M1 macrophages (E) and M2 macrophages (F) among CD11b^+^F4/80^+^ macrophages in each group (*n* = 6/group). (G, H) Statistical results of flow cytometric analysis of the frequencies of TNF‐α (G) and IL‐10 (H) secreted by CD11b^+^F4/80^+^ macrophages in each group (*n* = 6/group). The data are presented as the means ± SEMs. *p < 0.05, **p < 0.01, ***p < 0.001, ****p < 0.0001. BMDM, bone marrow‐derived macrophage; IL‐10, interleukin 10; LPS, lipopolysaccharides; SEM, standard error of the mean; TNF‐α, tumor necrosis factor‐α.

To investigate the anti‐inflammatory mechanisms of formoterol, we treated LPS‐induced BMDMs with formoterol and the GLS inhibitor BPTES. Compared with control treatment, formoterol treatment led to a significant decrease in M1 polarization and TNF‐α expression in BMDMs, whereas the expression of M2 polarization markers, such as CD206 and the anti‐inflammatory cytokine IL‐10 increased. However, these anti‐inflammatory effects of formoterol were abrogated upon cotreatment with the GLS inhibitor BPTES (Figure 7A–H). These results indicate that the ability of formoterol to reduce inflammation in BMDMs stimulated with LPS is likely due to its impact on GLS function.

## 4 Discussion

This study demonstrated that β2‐AR expression was reduced in MoMFs within the cardiac tissue of BALB/c mice with VMC, both in the early and peak stages of inflammation. In this model, stimulation of β2‐AR by formoterol ameliorated cardiac inflammation and fibrosis, and reduced the infiltration of CCR2^+^MHCII^high^ MoMFs in the heart. This effect was achieved through diminished infiltration of CCR2^+^ monocytes/macrophages. Mechanistically, formoterol enhanced GLS activity in macrophages under inflammatory conditions, thereby inhibiting macrophage polarization towards the M1 phenotype and suppressing the secretion of pro‐inflammatory cytokines. Therefore, stimulating β2‐AR in cardiac MoMFs could offer a new method for treating VMC.

β2‐AR agonists have demonstrated protective effects on heart failure and dilated cardiomyopathy. Clenbuterol, a β2‐AR agonist, enhanced heart function, decreased cell death, and maintained calcium balance in a rat model of ischemic heart failure [37]. Treatment with clenbuterol hydrochloride in a sheep model of right heart failure induced by pulmonary artery banding enhanced right ventricular function [38]. In rats with severe heart attack caused by tying off the coronary artery, β2‐AR agonists enhance measures of heart function, such as the left ventricular ejection fraction [39]. Clenbuterol treatment led to a notable enhancement in cardiac function for the majority of patients with nonischemic dilated cardiomyopathy necessitating left ventricular assist devices [40]. In our study using a mouse model of VMC induced by CVB3, we discovered that formoterol treatment effectively decreased heart inflammation and scarring while enhancing heart function during both the initial phase and increased inflammation. Currently, there are no specific treatments for VMC. Our study suggests a new therapeutic avenue for VMC treatment using β2‐AR agonists such as formoterol. This approach not only holds promise for mitigating cardiac damage but also for enhancing recovery, offering potential for clinical use.

Monocytes and macrophages are crucial for the development of inflammation in the immune response to heart damage. Upon cardiac damage, circulating monocytes expressing CCR2 accumulate around blood vessels [41], where Ly6C^high^CCR2^+^CX3CR1^low^ monocytes infiltrate and differentiate into macrophages within the injured area, contributing to the inflammatory response [42]. The increased presence of CCR2^+^MHCII^high^ macrophages in the heart primarily arises from the differentiation of circulating monocytes [43, 44]. This macrophage subtype exhibits phenotypic characteristics similar to those of classically described M1 macrophages and is associated with adverse pathogenic cardiac remodeling [42, 45]. β2‐AR is involved in controlling the activity of natural immune cells, changing their functional status when activated to produce anti‐inflammatory outcomes. Research has shown that stimulating macrophage β2‐AR induces a change towards an anti‐inflammatory state, preventing lipopolysaccharide (LPS)‐induced systemic inflammation and providing protection against renal ischaemia reperfusion injury [46]. In rat models of diabetes‐induced obesity, the activation of monocytes in the kidneys and heart, along with inflammatory and fibrotic responses, is decreased by β2‐AR agonists, providing defense against complications in the kidneys and heart caused by diabetes [7]. Electroacupuncture in a rat model of knee osteoarthritis led to elevated levels of synovial norepinephrine through β2‐AR, which suppressed excessive IL‐6 production in synovial macrophages by targeting the CXCL1‐CXCR2 pathway, ultimately relieving osteoarthritis [47]. Macrophages are essential for the development of CVB3‐induced myocarditis, particularly because different macrophage types can trigger conflicting inflammatory reactions [4]. In our BALB/c mouse model of VMC, similar to past research, we observed that β2‐AR agonists influence the transformation of innate immune cells and their function, leading to anti‐inflammatory effects. The β2‐AR agonist formoterol reduced the infiltration of MoMFs in the heart and decreased the proportion of Ly6C^high^ inflammatory monocytes in the blood. Formoterol inhibits the M1 polarization of MoMFs while encouraging M2 polarization, reducing the release of pro‐inflammatory cytokines and increasing the production of anti‐inflammatory factors. Furthermore, in a CVB3‐induced VMC mouse model, we demonstrated that the anti‐inflammatory effect of formoterol was mediated by inhibiting CCR2^+^ monocytes/macrophages via the use of a CCR2 inhibitor. Our research offers a fresh outlook on the impact of formoterol on myocarditis, suggesting that focusing on β2‐AR may be an effective approach for regulating immune reactions in inflammatory heart conditions.

Glutamine metabolism is closely linked with the phenotypic transformation and functionality of macrophages. Previous research has demonstrated that in an LPS‐induced BMDM M1 model, the use of the GLS inhibitor BPTES enhances the expression of M1 markers and the secretion of pro‐inflammatory cytokines. On the other hand, BPTES inhibits M2 marker expression and the release of anti‐inflammatory cytokines in a model of IL‐14‐induced BMDM M2 [22]. These results indicate that blocking the breakdown of glutamine encourages a shift towards the M1 phenotype, whereas transitioning to the M2 phenotype necessitates glutamine degradation. Our experiments conducted in a controlled environment showed that treatment of BMDMs with formoterol suppressed the expression of the M1 indicator CD86 and the release of the inflammatory cytokine TNF‐α, but enhanced the expression of the M2 indicator CD206 and the anti‐inflammatory cytokine IL‐10. However, the anti‐inflammatory effects of formoterol are negated when it is combined with the GLS inhibitor BPTES, indicating that the ability of formoterol to reduce inflammation may be due to its ability to promote the metabolic breakdown of glutamine. The potential mechanism by which formoterol affects glutamine metabolism in LPS‐induced BMDMs and thus mediates changes in phenotype and function could be explained as follows. Under normal conditions, glutamine is metabolized by GLS into glutamate, and glutamate is further deaminated to produce α‐ketoglutarate (αKG). αKG is subsequently involved in the tricarboxylic acid (TCA) cycle to undergo additional metabolism resulting in the production of CO2 and pyruvate [48, 49, 50]. During the M1 polarization process induced by LPS, transcriptional‐metabolic analysis revealed defects in the TCA cycle, possibly leading to inhibited glutamine hydrolysis [51, 52], thus reducing αKG production. The introduction of formoterol enhances GLS activity, thereby promoting the metabolic breakdown of glutamine and increasing αKG production. αKG enhances the activation of anti‐inflammatory genes by inducing epigenetic changes [19], leading to a shift in the phenotype and function of LPS‐stimulated BMDMs towards an anti‐inflammatory state. Though we investigated the effect of formoterol on GLS activity in vitro, the effect of formoterol on GLS activity in vivo may as an avenue for further study.

This study indeed has several limitations. First, we utilized a β2‐AR agonist for pharmacological intervention and examined its effects on VMC and cardiac MoMFs. Nevertheless, all pharmaceutical treatments lack specificity and may exist off‐target effects, leaving the potential for unintended consequences. Furthermore, the potential effects of β2‐AR agonist treatment on various nonmacrophage cells in the heart, including neutrophils, B cells, T cells, and cardiac fibroblasts, should not be disregarded. Further validation may require the specific knockout of β2‐AR in MoMFs combined with appropriate experimental designs. Third, the experiments conducted are limited to animal and cellular models, restricting the direct translation of findings to human clinical scenarios. Fourth, we did not set up a control group with formoterol treatment to assess the safety of formoterol to mice, however, the previous studies showed the safety profile of formoterol at a dose 0.3mg/kg [29] or 2mg/kg [53] to mice. Further research is needed to confirm whether the findings in mice regarding the effectiveness and safety of β2‐AR agonists also apply to patients with VMC and to investigate the additional mechanisms.

In summary, our study demonstrated that the β2‐AR agonist formoterol influences VMC progression by affecting the phenotypic transformation and functionality of MoMFs in a murine model. Additionally, our findings highlight the mechanism by which formoterol influences these changes, particularly through its impact on GLS activity in macrophages under inflammatory conditions. Thus, targeting β2‐AR could represent a promising treatment option for VMC.

## 5 Conclusion

Formoterol potentially serves as a significant metabolic regulator in the differentiation process of cardiac MoMFs, influencing this process by controlling GLS activity. Targeting β2‐AR exhibits potential as an effective approach for managing VMC. It is essential to acknowledge that these findings were derived under specific experimental conditions, with the current conclusions predominantly based on animal models. Future research is necessary to further investigate the feasibility of formoterol in clinical practice.

## Author Contributions

Quan‐liang Li was the main writer and had complete access to all the study data, assuming responsibility for the accuracy of the data analysis and the integrity of the data. Quan‐liang Li and Wei‐feng Wu were involved in the development of the study and planning. Ying‐xin Guo, Jing Qian, and Juan‐fen Li were involved in collecting and analyzing the data. Quan‐liang Li, Hua‐bao Xie and Ying‐xin Guo contributed to drafting the manuscript. Quan‐liang Li, Hua‐bao Xie and Ying‐xin Guo contributed to revision of the article and final approval.

## Ethics Statement

The Ethics Committee of the First Affiliated Hospital of Guangxi Medical University granted approval for this study (202306013).

## Conflicts of Interest

The authors declare no conflicts of interest.

## Data Availability

The corresponding author can provide the data supporting the findings of this study upon reasonable request. The article contains all the data and models created or utilized in the study.
